# Altered Lipid Profile in COVID-19 Patients and Metabolic Reprogramming

**DOI:** 10.3389/fmicb.2022.863802

**Published:** 2022-05-12

**Authors:** Tie Zhao, Chunhui Wang, Biyan Duan, Peipei Yang, Jianguo Wu, Qiwei Zhang

**Affiliations:** ^1^Guangdong Provincial Key Laboratory of Virology, Institute of Medical Microbiology, Jinan University, Guangzhou, China; ^2^Key Laboratory of Special Pathogen Prevention and Control of Hunan Province, Hengyang Medical College, Institute of Pathogenic Biology, University of South China, Hengyang, China; ^3^Department of Clinical Laboratory, Huizhou Central People’s Hospital, Huizhou, China; ^4^Foshan Institute of Medical Microbiology, Foshan, China; ^5^BSL-3 Laboratory (Guangdong), Guangdong Provincial Key Laboratory of Tropical Disease Research, School of Public Health, Southern Medical University, Guangzhou, China

**Keywords:** COVID-19, lipid, SARS-CoV-2, dyslipidemias, metabolic reprogramming

## Abstract

**Background:**

Coronavirus disease 2019 (COVID-19) is a global pandemic. Previous studies have reported dyslipidemia in patients with COVID-19. Herein, we conducted a retrospective study and a bioinformatics analysis to evaluate the essential data of the lipid profile as well as the possible mechanism in patients with COVID-19.

**Methods:**

First of all, the retrospective study included three cohorts: patients with COVID-19, a healthy population, and patients with chronic obstructive pulmonary disease (COPD). For each subject, serum lipid profiles in the biochemical data were compared, including triglycerides (TG), total cholesterol (TC), high-density lipoprotein cholesterol (HDL-C), and low-density lipoprotein cholesterol (LDL-C). Furthermore, bioinformatics analyses were performed for exploring the biological or immunological mechanisms.

**Results:**

In line with the biochemical data of the three cohorts, the statistical result displayed that patients with COVID-19 were more likely to have lower levels of TC and HDL-C as compared with healthy individuals. The differential proteins associated with COVID-19 are involved in the lipid pathway and can target and regulate cytokines and immune cells. Additionally, a heatmap revealed that severe acute respiratory syndrome coronavirus 2 (SARS-CoV-2) infections were possibly involved in lipid metabolic reprogramming. The viral proteins, such as spike (S) and non-structural protein 2 (Nsp2) of SARS-CoV-2, may be involved in metabolic reprogramming.

**Conclusion:**

The metabolic reprogramming after SARS-CoV-2 infections is probably associated with the immune and clinical phenotype of patients. Hence, metabolic reprogramming may be targeted for developing antivirals against COVID-19.

## Introduction

The infections of severe acute respiratory syndrome coronavirus 2 (SARS-CoV-2) led to the coronavirus disease 2019 (COVID-19) pandemic and have been a threat to public health across the world. There are 464,809,377 confirmed cases of COVID-19, including 6,062,536 deaths as of March 20, 2022 (World Health Organization [WHO], 2022). Public health and social and economic growth have been enormously influenced by the COVID-19 pandemic. SARS-CoV-2 contains four structural proteins, [envelope (E), membrane (M), nucleocapsid (N), and spike (S)], 16 non-structural proteins (Nsp1 to Nsp16), and eight accessory proteins (orf3a, orf6, orf7a, orf7b, orf8, orf9b, orf9c, and orf10). These proteins are involved in the viral life cycle and viral interaction with the host. Although the interaction between SARS-CoV-2 and the host is a moot point, the scientific community steadily gained an understanding of pathogenesis in the past.

Although the interactions between the immune system and lipid metabolism during SARS-CoV-2 infections remain unclear, the new development of tumor and metabolism study can provide new ideas and methods for the influence of SARS-CoV-2 infections. Interestingly, lipids are involved in viral pathogenesis and the pathophysiology of viral disease (Nie et al., 2020). Lipids not only constitute virus envelope but also involve viral replication and invasion. The composition of viruses and cells always includes lipids involved in membrane fusion and replication during the entry and the release from the host cell membrane. Previous studies (Wei et al., 2020; Jin et al., 2021; Mahat et al., 2021) have shown that lipid profiles, such as the total cholesterol (TC), high-density lipoprotein cholesterol (HDL-C), and low-density lipoprotein cholesterol (LDL-C), in patients with COVID-19 are significantly altered. TC increases in the cell membrane, which benefits the virus entry to the host cells and the membrane fusion (Theken et al., 2021). The alterations of lipid profile in patients with COVID-19 seem to be proportional to the clinical phenotype and might be a target for risk evaluation. In addition, TC can regulate T-cell-mediated immune response and constitute T-cell receptors (TCRs) as a critical regulator, directly or indirectly (Bietz et al., 2017; Puleston et al., 2020). A previous study about lymphocytic choriomeningitis virus (LCMV) also showed that the clearance of the LCMV was significantly delayed in hypercholesterolemic mice, and LCMV-specific CD8^+^ T cells were suppressed (Ludewig et al., 2001). Cholesterol accumulation reduced the activation of CD8α- dendritic cells, thereby impairing Th1 cell responses while enhancing Th2 cell responses (Kim et al., 2021). Other evidence from oncology has demonstrated that multiple lipid species can be sensed by innate immune cells including macrophages and dendritic cells. Dyslipidemia is a critical regulator of adaptive immunity, which in turn can regulate adaptive immune cells (Kim et al., 2021).

However, the concentration of lipid profiles in patients with COVID-19 was reported with variable values (Gao et al., 2020; Hu et al., 2020; Lei et al., 2020; Malik et al., 2021). A likely explanation is that the genetic phenotypes and underlying diseases are significantly different among patients with COVID-19. To extend the existing evidence regarding the relationship between COVID-19 and lipid profile, a retrospective study and mechanism exploration by bioinformatics analyses were performed. We did extensive research about the actual relation between viral pathogenesis and lipid alteration through existing data. We attempted to elucidate the correlation between lipid profile and immunoreaction among patients with COVID-19, including lipid metabolism and profile, for example, dyslipidemia mechanism, cytokines, and T-cell-mediated immune response.

## Materials and Methods

### Clinical Information

This retrospective study included three cohorts, 25 COVID-19 cases, 25 cases of the healthly examination population (control group, CG), and 25 cases with chronic obstructive pulmonary disease (COPD), recruited from the Huizhou Central People’s Hospital. COPD and CG never went through a previous infection with COVID-19 or received the vaccination. The patients were diagnosed with COVID-19 in light of the World Health Organization (WHO) guidelines.^1^ The nasopharyngeal swabs of patients with COVID-19 were collected for diagnosis. The laboratory-confirmed patient was defined as a positive result on the real-time reverse-transcriptase polymerase chain reaction (RT-PCR) assay of nasopharyngeal swab specimens. These cases were well balanced for gender, age, and primary disease. All the COVID-19 symptoms were mild, and no severe cases appeared. This study was performed according to the principles of the Declaration of Helsinki and approved by the Huizhou Central People’s Hospital following its guidelines for the protection of individual privacy.

### Biochemical Measurements

For three cohorts, serum lipid profiles of patients with COVID-19, patients with COPD, and the healthy examination population were tested by biochemical methods (Roche Cobas 8000), including triglycerides (TG), TC HDL-C, and LDL-C. The sera of patients with COVID-19 were collected on admission.

### Dataset Collection

The data based on the initial screening are retrieved mainly from the National Center for Biotechnology Information. Proteomic and lipidomic data from the sera/plasma of patients with COVID-19 were acquired from the early studies (Shen et al., 2020; Wu et al., 2020) and GEO datasets (GEO accession number: GSE157103). R was used to screen for differential proteins and lipids (R Core Team, 2018). Glycolysis pathway data were acquired from Caccuri et al. (2021). Profiles of serum cytokines and chemokines in patients with COVID-19 were acquired from Zawawi et al. (2021). SARS-CoV-2 S, E, Nsp15, Nsp16 (Sharma et al., 2021), and Nsp2 (Davies et al., 2020) proteins were determined and compared with the host transcriptomic responses to key viral genes.

### Bioinformatics Analyses

The Venn diagram was generated based on the datasets (Venn, 2022). Gene Ontology (GO) [involving biological process (BP), cell component (CC), molecular function (MF), and Kyoto Encyclopedia of Genes and Genomes (KEGG)] were utilized to analyze the expected signaling pathways and corresponding functions of differential proteins via the package of R or platform of Enrichr (Chen et al., 2013; R Core Team, 2018). The heatmap was also generated by the package of R (R Core Team, 2018). The proteins network was constructed *via* the STRING dataset (Szklarczyk et al., 2021). Lipidmap was produced by the analysis of KEGG (Fahy et al., 2007). CytoHubba, an app of Cytoscape, was screened for hub genes (Shannon et al., 2003). The immune cell infiltration analysis was performed by GEPIA2021 (Li et al., 2021).

### Statistical Analysis

Values of serum lipid were shown as the mean (M) ± standard deviation (*SD*). The comparison for the lipid of three cohorts was made by one-way ANOVA using the SPSS 24.0 (IBM Corp.). The least significant difference (LSD) was further compared to show any significant difference between the two groups. Graphic plotting was generated using GraphPad Prism 8 (GraphPad Software, Inc.).

## Results

### Baseline Characteristics of Coronavirus Disease 2019 Patients

The study included 75 cases, which consisted of 25 COVID-19 patients positive for SARS-CoV-2 RNA, 25 cases diagnosed with COPD that all the history and symptoms supported, and 25 healthy people. The mean ages of CG, patients with COVID-19, and patients with COPD were 51 ±16.2, 47 ±15.4, and 54 ±17.6 years, respectively. The patients with COVID-19 did not use any statins according to medication guidelines. Other cohorts were similar. In all cases, previously diagnosed metabolic diseases (obesity, hypertension, and diabetes) were not incorporated based on self-report.

### The Lipid Level Change During the Coronavirus Disease 2019 Courses

**FIGURE 1:**
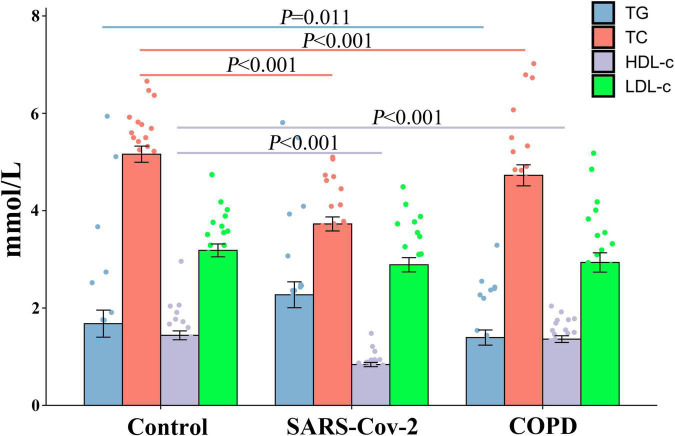
Comparison of the lipid level between the COVID-19, COPD patients, and healthy population. In COVID-19 and COPD patients, TG and HDL-C expression was reduced, but not for TC and LDL-C.

We found that there were significant differences in TG (*F* = 3.506, *P* < 0.05), TC (*F* = 17.123, *P* < 0.0001), and HDL-C (*F* = 21.473, *P* < 0.0001) levels between the three cohorts, but no significant difference was observed in LDL-C (*F* = 0.97, *P* > 0.05). In line with the biochemical data of the three cohorts, the statistical result displayed that the patients with COVID-19 were more likely to have a lower level of TC (*P* < 0.001) and HDL-C (*P* < 0.001) as compared with the healthy examination population. Patients with COPD had similar results (*P* < 0.001) (Figure 1).

### Differential Proteins and Sub-Network Module Enrichment Analysis

**FIGURE 2:**
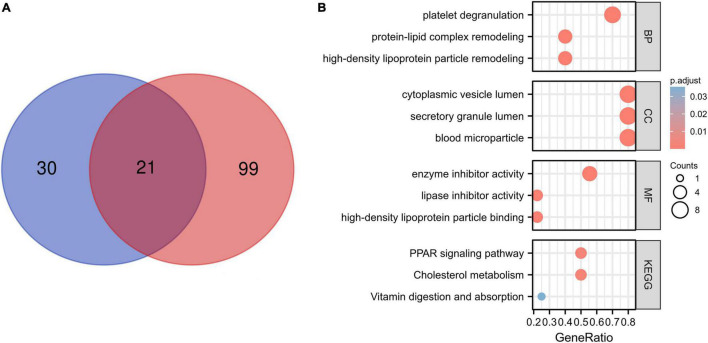
Proteome of the patients with COVID-19 and enrichment analysis. **(A)** A total of 21 common proteins were identified according to the proteome of the sera and plasma from patients with COVID-19 by Venn diagram. The blue color shows the proteome of sera of patients with COVID-19, and the red color shows plasma proteome associated with COVID-19. **(B)** A total of 21 common proteins involved in lipid pathway by the enrichment analysis, for example, protein–lipid complex remodeling and high-density lipoprotein particle remodeling.

Differential proteins in patients with COVID-19 versus CG were acquired in GEO datasets. To further study the role of differential proteins, GO and KEGG signaling pathway analysis indicated that some proteins were involved in lipid pathways (Supplementary Table 1), such as the PPAR signaling pathway, cholesterol metabolism, fatty acid biosynthesis, positive regulation of cholesterol esterification (GO:0010873), and high-density lipoprotein particle remodeling (GO:0034375). The proteome of the sera/plasma of patients with COVID-19 showed 21 common proteins (Figure 2A), which are ORM1, ITIH3, ALB, SAA2, PGLYRP2, APOA1, NID1, GSN, CPN2, LGALS3BP, AGT, LCP1, C2, CLEC3B, ITIH4, APOM, CRTAC1, APOA2, ORM2, AHSG, and GPLD1. These 21 proteins had similar enrichment results that were associated with lipid pathways (Figure 2B and Supplementary Table 2). The lipid pathway included cholesterol metabolism, the PPAR signaling pathway, fat digestion and absorption, positive regulation of cholesterol esterification (GO:0010873), regulation of cholesterol esterification (GO:0010872), and so on. Conversely, lipid composition in patients with COVID-19 did not correspond with what other studies showed (data not shown). The enrichment analysis of lipidome in patients with COVID-19 showed that the blood lipid of humans was mainly involved in sphingolipid metabolism, cholesterol metabolism, fat digestion and absorption, and the sphingolipid signaling pathway (Supplementary Table 3).

### Severe Acute Respiratory Syndrome Coronavirus 2 Infection and Viral Proteins Cause Metabolic Reprogramming

**FIGURE 3:**
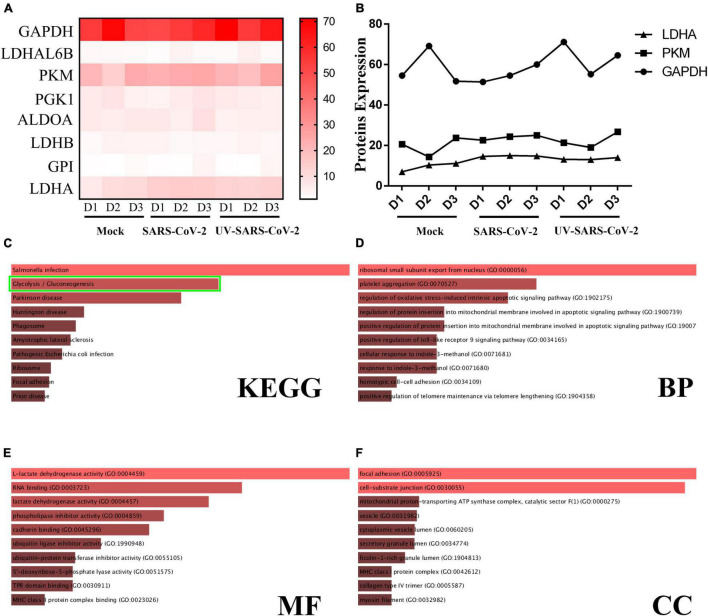
SARS-CoV-2 infection upregulates the glycolysis pathway. **(A)** A heatmap was generated from the SARS-CoV-2 infection and UV-inactivated SARS-CoV-2 treatment. In both groups, LDHA level was increased. **(B)** The expression of LDHA, PKM, and GAPDH was significantly different among the healthy population, SARS-CoV-2, and UV-SARS-CoV-2 groups. **(C)** The KEGG analysis revealed the SARS-CoV-2 infection and protein correlation involved in the glycolysis pathway (the green bounding box). **(D–F)** The BP, MF, CC analysis revealed the SARS-CoV-2 infection involved in the oxidative stress, the lactate dehydrogenase activity, et al.

The heatmap analysis showed the expression and increment of LDHA, GAPDH, and PKM post-SARS-CoV-2 infection; UV-inactivated SARS-CoV-2 can increase LDHA (Figures 3A,B). The GO and KEGG signaling pathway analysis indicated that the differential proteins of SARS-CoV-2 in endothelial cells were involved in glycolysis (Figure 3C). The lipid pathway and glycolysis occurred showed the potential for metabolic reprogramming post-SARS-CoV-2 infection (Figures 3D–F).

### Protein Network for Targeting Cytokine and Chemokine Regulation

**FIGURE 4:**
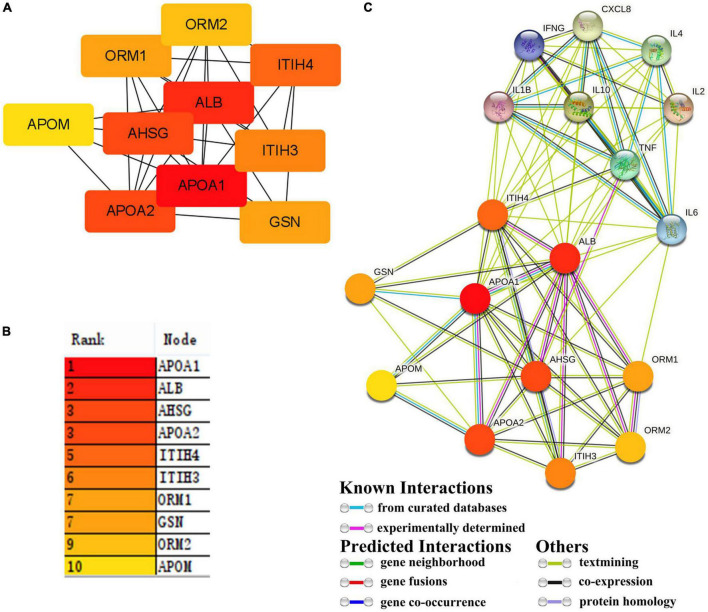
Hub protein in 21 proteins and interaction between hub proteins and cytokines. **(A,B)** 21 proteins were screened for 10 hub proteins. **(C)** The interaction between hub proteins and cytokines.

The first 10 nodes (subproteins) (APOA1, ALB, AHSG, APOA2, ITIH4, ITIH3, ORM1, GSN, ORM2, and APOM) with the highest values were screened as fibrin clot (clotting cascade) and lipoprotein particle (Figures 4A,B). A protein–protein interaction (PPI) network for the first 10 nodes was constructed using the STRING database. These subproteins may regulate cytokines and chemokines in patients with COVID-19 (Figure 4C).

### Ten Sub-Proteins and Cell Type-Level Expression Analysis

**FIGURE 5:**
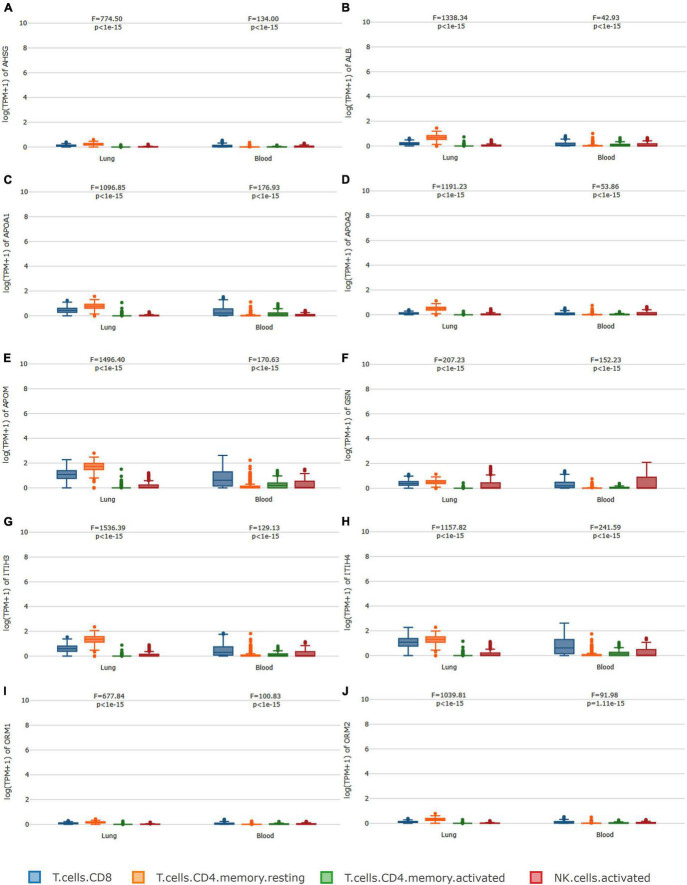
Immune cell type-level expression analysis. In regard to **(A–J)**, the analysis of immune infiltration revealed that the CD4^+^ cell has the highest median value in the lung, and the CD8^+^ cell has the highest median value in blood except for GSN. A component analysis of the immune cells showed that CD4^+^ T cells, CD8^+^ T cells, and NK cells were significantly related to the 10 subproteins.

GEPIA2021 analysis further confirmed the correlations between the 10 sub-protein levels and cell types. Regarding the 10 sub-protein expression levels, the analysis of immune infiltration revealed that the CD4^+^ cell has the highest median value in the lung and the CD8^+^ cell has the highest median value in blood except GSN. A component analysis of the immune cells showed that CD4^+^ T cells, CD8^+^ T cells, and NK cells were significantly related to 10 subproteins (Figure 5).

### The Viral Proteins Correlate With Metabolic Reprogramming

**FIGURE 6:**
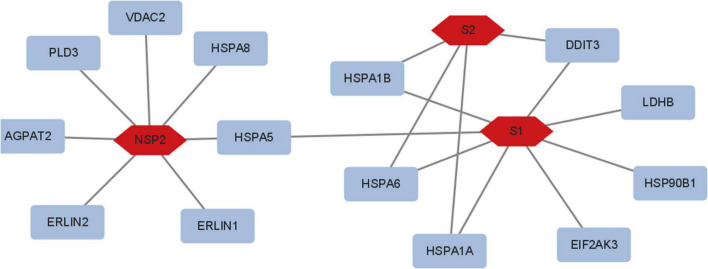
The viral proteins involved in metabolic reprogramming. S1 regulated HSPA1A, HSPA6, HSPA1B, DDIT3, LDHB, HSP90B1, and EIF2AK3; S2 regulated HSPA1B, HSPA1A, HSPA6, and DDIT3; Nsp2 regulated PLD3, VDAC2, HSPA8, HSPA5, ERLIN1, ERLIN2, and AGPAT2. N, Nsp15, and Nsp16 did not modify metabolic reprogramming.

The S and Nsp2 proteins may involve metabolic reprogramming (Figure 6), but N, Nsp15, and Nsp16 would not. S1 subunit seems to regulate HSPA1A, HSPA6, HSPA1B, DDIT3, LDHB, HSP90B1, and EIF2AK3; S2 subunit seems to regulate HSPA1B, HSPA1A, HSPA6, and DDIT3; Nsp2 may regulate PLD3, VDAC2, HSPA8, HSPA5, ERLIN1, ERLIN2, and AGPAT2. These proteins are related to glycolysis and lipid pathways.

## Discussion

Lipid profile alteration was used as a potential biomarker to aid diagnostics via triggers of viral infection. Our findings indicate that TC and HDL-C were reduced in patients with COVID-19, but TG and LDL-C did not. This finding is consistent with previous studies (Li G. et al., 2020; Li J. et al., 2020; Lv et al., 2020; Sun et al., 2020; Tanaka et al., 2020; Xie et al., 2020; Xue et al., 2020; Zhang B. et al., 2020; Zhang Q. et al., 2020). The serum/plasma concentrations of total TC and HDL-C were significantly lower in patients with COVID-19 with more severe diseases but were not for the TG. Significant changes in host lipidomes were observed in the cases of viral infection with severe disease, which induced changes in host immune function and benefited viral replication. On account of population and deviation, the results are possibly different in LDL-C. Distinctly, the heterogeneity between studies was generally large-to-extreme and multiple studies included small sample sizes. Interestingly, TC and HDL-C levels were associated with the clinical phenotype of SARS-CoV-2 infection. TC and HDL-C have beneficial effects on various pulmonary diseases and other diseases (Nie et al., 2020) and play a key role in modulating both innate and adaptive immune cell responses (Bietz et al., 2017). HDL has a function in inducing an anti-inflammatory or inflammatory profile (Van Lenten et al., 1995; Khovidhunkit et al., 2004). The reduced plasma levels of HDL can be found in patients with infection and sepsis (Wu et al., 2004; Cirstea et al., 2017). Lipid profile alteration is a useful indicator for early warning of the severity of COVID-19 disease (mild or severe) (Nie et al., 2020).

Energy and metabolites are required for cell survival. SARS-CoV-2 infections lead to a hypoxic microenvironment (Bhattacharya et al., 2021). This process is akin to the tumor microenvironment (TME) that the feature of TME is hypoxic. This promotes the host to compensate for their metabolic profiles to sustain a reprogramming state. The heatmap analysis showed that the expression of LDHA, a protein involved in glycolysis, was increased in SARS-CoV-2 infection. Furthermore, the proteomic data in two studies were analogous (Shen et al., 2020; Wu et al., 2020). The interaction network and enrich analysis revealed that the related pathways of lipid were located in the central node of all patient groups. There were significant similarities in lipid pathways among patients with COVID-19 from different regions. The 21 common proteins in this study supported this view. Enrichment analysis showed that the proteins were mainly involved in cholesterol metabolism, the PPAR signaling pathway, and so on. Anyhow, the hypoxic microenvironment in patients with COVID-19 increases the metabolic reprogramming for local nutrients and oxygen. However, the exact role or influence of metabolism reprogramming in SARS-CoV-2 immune response remains unclear, and lipids may regulate SARS-CoV-2 infection by multiple mechanisms.

Mechanisms derived from the previous study may also shed light on factors contributing to SARS-CoV-2 infectivity, where cholesterol is important either through immune regulator or by mediating signal pathway. The effects of lipids on infection development play a pivotal role. The function of lipids was gradually decrypted, which was used as an alternative source in pathologic conditions (Olsen et al., 2021), and was involved in the virus infection, was involved in transport of cell membrane, and activated intricate signaling pathways related to the immune system (Yu et al., 2021). Lipid metabolism dysfunction in the host has extensive effects on immune cells. The hub proteins were correlated with cytokines and chemokines in patients with COVID-19, and a distinct connection with immune cells was identified. However, the explanations for the lipid phenotype of patients are complex. In addition, individuals with underlying comorbidities (primary disease and metabolic disturbance in patients) will have more dramatic changes such that cholesterol provides a more complicated explanation and elaborate medical regimen. Due to the complex composition of lipids and a dynamically anabolic process, different points-in-time may respond very diversely to changes in lipid metabolism and give rise to ambiguous phenotypes. Enrichment analysis of lipids showed blood lipids of humans mainly involved in the sphingolipid metabolism, the cholesterol metabolism, fat digestion and absorption, and the sphingolipid signaling pathway, which suggested that the pathway was mainly for lipid-controlled biosynthesis or signaling. However, the studies of blood lipid were very heterogeneous. The quantification of blood lipids is still non-determined because of the complex component and much fluctuation of lipid quality and quantity in different space and time.

Interestingly, the lipid levels in patients with COPD changed and compared with the healthy population, but it was similar to patients with COVID-19. In addition, hypoxia is a common characteristic of patients that can change the metabolism (Grieb et al., 2021; Palm and Ekström, 2021). Hence, the patients were artificially ventilated, a procedure that can cause intraoperative complications but also can remit glycolysis or further metabolic reprogramming. So, the external reason was partially confirmed. The glycolysis suppression may be taken as a strategy for COVID-19 therapy and has profound therapeutic implications and significance. On the other hand, the lipid metabolism in patients with COVID-19 as a major altered function is highly similar to infection and sepsis, which is in accordance with a reply for multiple pathogens infection and in modulating inflammatory responses by the lipid moieties. These results indicate that metabolism plays a key role in SARS-CoV-2 pathogenesis and is a possible therapeutic target.

Meanwhile, the data showed that LDHA expression is increased in UV-SARS-CoV-2 infection. Besides anoxia, metabolic reprogramming was induced by the viral proteins as well. Viral structural proteins are involved in such processes. Numerous viruses (Negro, 2010; Funderburg and Mehta, 2016; Melo et al., 2016; Tisoncik-Go et al., 2016; Eisfeld et al., 2017; Kyle et al., 2019), such as Ebola virus, HIV, HBV, HCV, and homologous SARS-CoV (Wu et al., 2017) and MERS (Yan et al., 2019), can dramatically alter the human plasma lipidome. Even in the 12 years since the SARS-CoV infection, lipidome had been significantly changed (Wu et al., 2017). Therefore, viral proteins are involved similarly in metabolic reprogramming.

The viral proteins (Nsp2 and S) also affect lipid synthesis and modification (Díaz, 2020). The S protein of SARS-CoV-2 is a key protein. Numerous studies have confirmed that the S protein binds to ACE2 receptors on the surface of host cells to facilitate viral entry (Du et al., 2009; Walls et al., 2020). The S protein comprises S1 and S2 subunits in the virus replication cycle, binding the host cell receptor or fusing the viral envelope with host cell membranes. S1 plays an important role in protein processing in the endoplasmic reticulum, lipid, atherosclerosis, and so on. S2 was concerned with protein processing in the endoplasmic reticulum, the lipid, and atherosclerosis. Therefore, both S1 and S2 also modify lipid synthesis. Nsp2, a non-structural protein of SARS-CoV-2, disrupts host cell cycle and has similar functionality, which was concerned with protein processing in the endoplasmic reticulum, the lipid, and atherosclerosis. Our analysis revealed that S and Nsp2 proteins are associated with HSPA5, HSPA6, and LDHB in metabolic reprogramming. The hub proteins do not overlap, so SARS-CoV-2 pathogenesis is complicated. These findings also suggest an unknown potential protein inducing lipid synthesis and modification. What is driving the metabolic reprogramming is not clear.

In this study, essential baseline data, such as primary disease and statin use or not, might eliminate the observed heterogeneity. However, the exact timing of the blood collection for lipid profile remains uncertain. However, this can be ignored, as lipid metabolism of SARS-CoV-2 infection is a lengthy process as stated earlier. To eliminate the large between-study heterogeneity, population experiments have been incorporated in this study. This study mainly discussed pivotal lipids (TC and HDL-C) and glycolysis in metabolic reprogramming but did not mention other lipid species, such as the sphingolipids, and their related pathways, or amino acids, organic acids, and nucleotides. In addition, the full impact of metabolic reprogramming in SARS-CoV-2 infection cannot be confirmed by cell culture without pressures imposed by the immune microenvironment.

## Conclusion

In conclusion, viral infection induces the alteration of host metabolic reprogramming, which is a remarkable feature. This alteration not only changes the immune and clinical phenotype of patients but is also involved in viral pathogenesis. So the virus–host interaction is figured thoroughly out. Therefore, antivirals may be developed *via* further study of the metabolic reprogramming mechanism along with the key proteins.

## Data Availability Statement

The original contributions presented in the study are included in the article/Supplementary Material, further inquiries can be directed to the corresponding author/s.

## Ethics Statement

The studies involving human participants were reviewed and approved by the Huizhou Central People’s Hospital. Written informed consent for participation was not required for this study in accordance with the national legislation and the institutional requirements.

## Author Contributions

TZ wrote the manuscript and created the figures. CW provided the data of serum of patients with COVID-19. BD and PY edited the manuscript. JW and QZ reviewed the data and revised the manuscript. All authors contributed to the article and approved the submitted version.

## Conflict of Interest

The authors declare that the research was conducted in the absence of any commercial or financial relationships that could be construed as a potential conflict of interest.

## Publisher’s Note

All claims expressed in this article are solely those of the authors and do not necessarily represent those of their affiliated organizations, or those of the publisher, the editors and the reviewers. Any product that may be evaluated in this article, or claim that may be made by its manufacturer, is not guaranteed or endorsed by the publisher.

## Footnotes

1
https://extranet.who.int/pqweb/vitro-diagnostics/coronavirus-disease-covid-19-pandemic-%E2%80%94-emergency-use-listing-procedure-eul-open
